# Timing of antibiotic therapy in the ICU

**DOI:** 10.1186/s13054-021-03787-z

**Published:** 2021-10-15

**Authors:** Marin H. Kollef, Andrew F. Shorr, Matteo Bassetti, Jean-Francois Timsit, Scott T. Micek, Andrew P. Michelson, Jose Garnacho-Montero

**Affiliations:** 1 Division of Pulmonary and Critical Care Medicine, Washington University School of Medicine, 660 South Euclid Avenue, MSC 8052-43-14, St. Louis, MO 63110 USA; 2 Pulmonary and Critical Care Medicine, Medstar Washington Hospital, Washington, DC USA; 3 Infectious Diseases Unit, Department of Health Sciences, San Martino Policlinico Hospital - IRCCS, University of Genoa, Genoa, Italy; 4 AP-HP, Bichat Claude Bernard Hospital, Medical and Infectious Diseases ICU (MI2), IAME, INSERM, Université de Paris, Paris, France; 5 Department of Pharmacy Practice, University of Health Sciences and Pharmacy, St. Louis, MO USA; 6 Intensive Care Clinical Unit, University Hospital Virgen Macarena, Seville, Spain

**Keywords:** Antibiotics, Sepsis, Pneumonia, Timing, Outcomes

## Abstract

Severe or life threatening infections are common among patients in the intensive care unit (ICU). Most infections in the ICU are bacterial or fungal in origin and require antimicrobial therapy for clinical resolution. Antibiotics are the cornerstone of therapy for infected critically ill patients. However, antibiotics are often not optimally administered resulting in less favorable patient outcomes including greater mortality. The timing of antibiotics in patients with life threatening infections including sepsis and septic shock is now recognized as one of the most important determinants of survival for this population. Individuals who have a delay in the administration of antibiotic therapy for serious infections can have a doubling or more in their mortality. Additionally, the timing of an appropriate antibiotic regimen, one that is active against the offending pathogens based on in vitro susceptibility, also influences survival. Thus not only is early empiric antibiotic administration important but the selection of those agents is crucial as well. The duration of antibiotic infusions, especially for β-lactams, can also influence antibiotic efficacy by increasing antimicrobial drug exposure for the offending pathogen. However, due to mounting antibiotic resistance, aggressive antimicrobial de-escalation based on microbiology results is necessary to counterbalance the pressures of early broad-spectrum antibiotic therapy. In this review, we examine time related variables impacting antibiotic optimization as it relates to the treatment of life threatening infections in the ICU. In addition to highlighting the importance of antibiotic timing in the ICU we hope to provide an approach to antimicrobials that also minimizes the unnecessary use of these agents. Such approaches will increasingly be linked to advances in molecular microbiology testing and artificial intelligence/machine learning. Such advances should help identify patients needing empiric antibiotic therapy at an earlier time point as well as the specific antibiotics required in order to avoid unnecessary administration of broad-spectrum antibiotics.

## Background

Infections are among the most common indications requiring care in an intensive care unit (ICU). The Extended Study on Prevalence of Infection in intensive Care III (EPIC III) was a recent international point prevalence study examining the occurrence of infections in ICUs [1]. Among 15,165 qualifying patients, 8135 (54%) had at least one suspected or proven infection on the study day and 1921 (24%) of these patients had more than one suspected or proven infection. Interestingly, multilevel analysis demonstrated that infection with antibiotic-resistant pathogens including vancomycin-resistant *Enterococcus* (VRE), *Klebsiella* species resistant to β-lactams, or carbapenem-resistant *Acinetobacter* species were associated with a higher risk of in-hospital death compared to susceptible microorganisms [1]. Escalating antimicrobial resistance for all pathogen types (bacterial, fungal, viral) has also increasingly impacted the outcomes of critically ill patients as suggested by EPIC III and other studies. The World Health Organization considers antimicrobial resistance to be a major threat to human health and a recent Wellcome Trust report suggests that nearly 300 million individuals will die over the next several decades as a direct result of antimicrobial resistance [2, 3]. Similarly, in the United States antibiotic resistant pathogens cause more than 2 million infections and 23,000 deaths per year as reported by the Centers for Disease Control and Prevention [4].

**Fig. 1:**
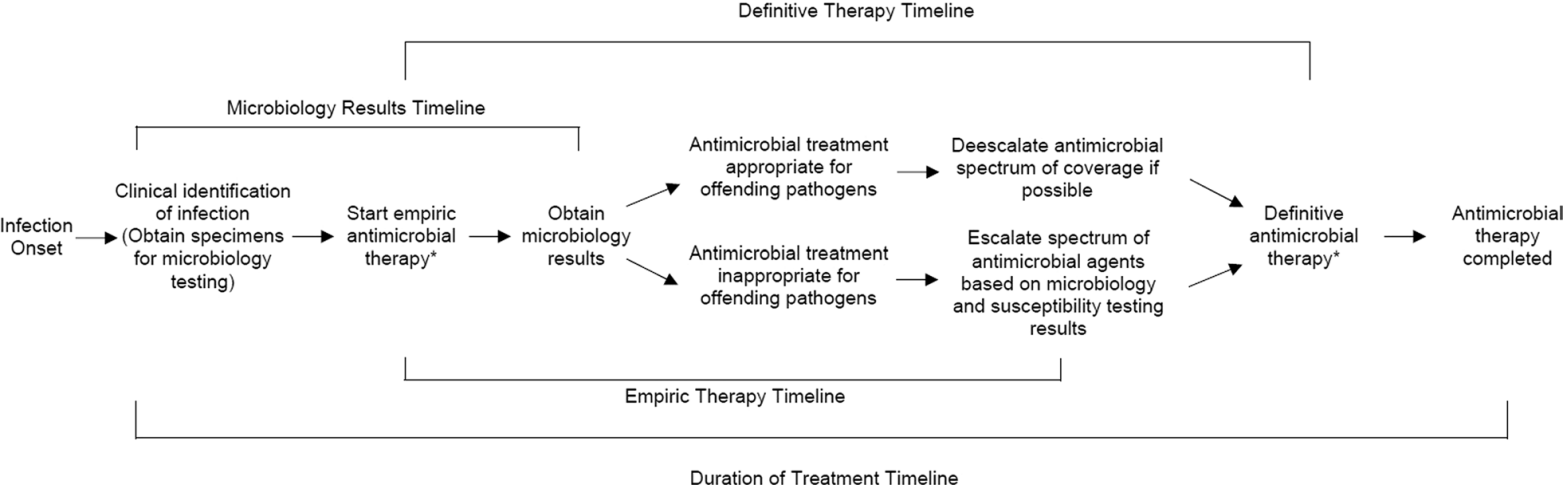
Important antibiotic related timelines potentially impacting the outcomes of infected critically ill patients. *Prolonged infusion duration of antimicrobials to increase antimicrobial drug exposure for the offending pathogen

Given the common occurrence of infections in the ICU, along with escalating antimicrobial resistance, we set out as our main goal to review the available literature regarding the importance of time related variables impacting antibiotic therapy (Fig. 1). We also wanted to provide some “common sense” recommendations supported by published evidence that may help clinicians optimize antibiotic therapy for critically ill patients and potentially improve their outcomes while minimizing further resistance emergence.

### Timing of appropriate therapy—septic shock

**Fig. 2:**
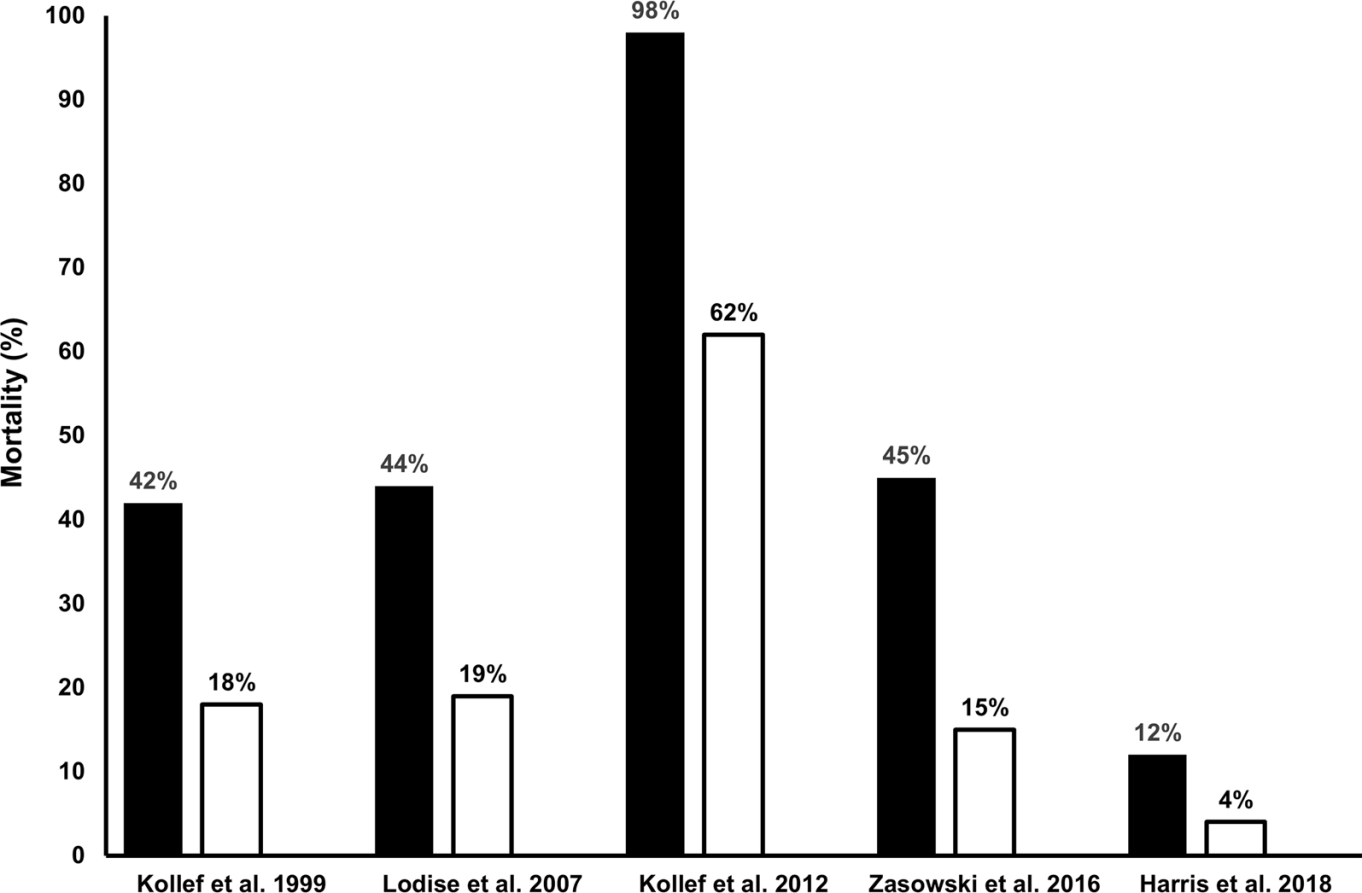
Bar graph depicting mortality for patients receiving delayed appropriate antibiotic therapy (black bars) and those receiving timely appropriate antibiotic therapy (white bars). See references 5–9 for individual study characteristics

Although controversy persists regarding many aspects of care for septic patients, nearly all agree that timely and appropriate antibiotic treatment is a necessary first step to insure good outcomes (Fig. 2) [5–9]. Interest in the issue of appropriate antibiotic treatment arose over two decades ago [5]. For example, Kumar and colleagues documented that for each hour delay in the administration of appropriate antibiotic(s) the patient’s risk for death increased substantially [10]. These authors demonstrated that for every hour’s delay until appropriate antibiotic administration led to a more than 10% increase in crude mortality. Specifically, if one did not begin appropriate therapy within 1 h of shock, the odds ratio (OR) for mortality increased from 1.67 in hour 2 to 92.54 with delays > 36 h [10]. Subsequent analyses examining the value of care bundles in sepsis confirmed the crucial importance of timely antimicrobials and source control [11]. A review of over 1000 patients with septic shock arising from Gram-negative pathogens revealed that inappropriate antibiotic therapy (identified based on the failure to administer an in vitro active antibiotic within six hours) independently increased the risk for mortality nearly fourfold [12].

Despite multiple analyses emphasizing the need for appropriate antibiotic treatment, documented rates of appropriate therapy in chart audits have not improved. Some suggest that relying on ORs to describe the burden of inappropriate therapy have not sufficiently motivated clinicians to change behavior. Therefore, Vazquez-Guillamet et al. shifted the emphasis from reliance on ORs to making the burden of inappropriate therapy much more tangible for the bedside provider. Specifically, they determined the number needed to treat (NNT) with appropriate therapy to save one life [13]. In over 1000 subjects with septic shock caused by a range of pathogens these investigators calculated that appropriate therapy enhanced the likelihood of survival at least threefold. More importantly, this converted into a NNT to save one life of only 5 [13]. A recent meta-analysis of the import of appropriate antibiotic therapy in a range of infectious nicely summarizes how the value of appropriate therapy increases in parallel with a patient’s severity of illness. Bassetti and colleagues identified 114 studies of appropriate therapy, 63 of which specifically dealt with sepsis and septic shock. The strongest positive impact of appropriate antibiotic treatment was observed among those with septic shock [14]. Appropriate therapy in sepsis not only significantly reduced in-hospital mortality (OR 0.44, 95% Confidence Interval 0.37–0.52) but also reduced length of stay by approximately 5 days [14]. That one can reduce both rates of death while simultaneously improving resource use and throughput underscores the true significance of this aspect of sepsis care.

Why does inappropriate therapy persist in clinical practice? In part, there may be delayed recognition of sepsis. More likely, the issue lies with the clinician. Looking at the factors associated with inappropriate therapy, the strongest variable related to failure to prescribe appropriate therapy relates to the prescriber not considering the presence of antibiotic resistance. With escalating rates of antibiotic resistance, the strongest factor independently associated with inappropriate therapy has been infection due to a resistant pathogen. In other words, the central factor propelling inappropriate therapy is failure to realize a patient’s risk factors for infection with an antibiotic resistant pathogen.

### Appropriate therapy optimization—bacterial infections

When initiating antimicrobial treatment in ICU patients, the choice of agents is most often empirical based on the site of infection, clinical severity and patient comorbidities [15]. Another key element for guiding appropriate empirical therapy is identifying risk factors for infection with multidrug-resistant bacteria (MDRB), so as to rationalize the empirical use of broad-spectrum antibiotics and prevent their unnecessary utilization. Recent literature suggests that initial antimicrobial therapy that is too broad is associated with poor outcomes. Webb et al. examined 1995 patients with community acquired pneumonia of whom 39.7% received broad-spectrum antibiotics, but MDRB were recovered in only 3% [16]. Broad-spectrum antibiotic treatment was associated with an increased mortality risk even after adjusting for prognostic covariates. Antibiotic-associated events were found in 17.5% of dying patients in the broad-spectrum group and may explain in part the worse outcomes for this cohort. The absence of bacteriological documentation in the majority of patients receiving broad-spectrum therapy suggests that other disease processes mimicking pneumonia and requiring alternative treatments may also have been missed [16].

Rhee et al. conducted a multicenter cohort study of 17,430 adults with sepsis and positive clinical cultures [17]. Among the 15,183 cases where antibiotic susceptibility testing was available, 12,398 (81.6%) received appropriate antibiotics. Less than 30% of cases were due to MDRB. Unnecessarily broad-spectrum treatment (defined as coverage of methicillin-resistant *Staphylococcus aureus*, VRE and ceftriaxone-resistant Gram-negative bacteria (GNB) when none of these were isolated) occurred in 8405 (67.8%) cases. The adjusted odds ratio for in-hospital death was 1.27 (1.06–1.4) when comparing unnecessarily broad-spectrum and not unnecessarily broad-spectrum initial antibiotic therapy. Unnecessarily broad antibiotic therapy was also associated with increases in acute kidney injury and *Clostridium difficile* infections.

Although it is difficult to ascertain with certainty the presence of an MDRB infection before pathogen identification and susceptibility testing, several factors can help clinicians in guiding broad-spectrum therapy [18, 19]. The conditions that influence risk for MDRB infection include recent hospitalization, prior antibiotic exposure, hospital- or healthcare-associated infection, known colonization with MDRB pathogens and local hospital and ICU epidemiology [18, 19]. However, none of these risk factors are completely accurate and the fear of bacterial resistance often drives overuse of broad-spectrum antimicrobials.

In patients colonized with extended-spectrum beta-lactamase (ESBL) producing GNB, carbapenem use increased from 69 to 241 per 1000 patient-days in patients who will not develop an ESBL infection and only 7.5% of infection-related ventilator-associated complications could be attributed to ESBL GNB in ESBL colonized patients [20, 21]. Among patients colonized with ESBL GNB, the site of colonization and its quantitative assessment may help to predict ESBL infections [22]. Similarly, MRSA colonization has been shown to increase empiric vancomycin use by 3.3 fold even in the absence of infection that would justify vancomycin use [23]. The use of rapid molecular tests (genotypic or phenotypic) to identify microorganisms and resistance mechanisms will probably help to increase the likelihood that empirical therapy is also definitive therapy (Fig. 1) while also avoiding unnecessary antibiotic exposures. Turn-around-time of these techniques is less than 2–4 h for routine use and will likely be reduced to less than 1 h in the near future [24, 25].

Besides the use of broad-spectrum antibiotics, combination antibiotic regimens (mostly a pivotal beta-lactam and an aminoglycoside) can help provide appropriate initial coverage while avoiding the systematic use of empiric carbapenems, providing that the patient is at low risk of infection with ESBL GNB [9]. The beneficial effect of dual antibiotic therapy is debated and probably most useful in neutropenic patients and infections due to difficult-to-treat GNB such as *Pseudomonas aeruginosa* [26, 27].

### Appropriate therapy optimization—fungal infections

There is considerable clinical evidence that delayed initiation of appropriate treatment is associated with increased mortality in patients with invasive fungal infections (IFI) [7, 28–30]. This is especially the case for critically ill patients with candidemia and septic shock [7, 31–33]. Although a specific cut-off point has not been established, several retrospective studies generally support the view that early and effective antifungal therapy is important for survival of patients with IFIs [29, 30]. Specifically, in a retrospective analysis of 157 candidemic patients, Morrell and colleagues found that, the administration of antifungal treatment ≥ 12 h after the collection of the first blood culture positive for *Candida* was an independent risk factor for hospital mortality (OR 2.09) [29]. Similarly, in another retrospective study of 230 candidemic patients, mortality was lowest (15%) when fluconazole therapy was started on the same day the blood culture was performed and rates rose progressively with time to initiation of fluconazole [30]. Another study of 446 patients showed significant mortality benefit when antifungal treatment was administered within 72 h of a positive blood culture for *Candida* [34].

The findings that a delay in initiating appropriate treatment is associated with increased mortality [29, 30], has contributed to recent guidelines recommending initiation of empirical antifungal therapy in critically ill septic patients at high risk for IFI [35]. Nonetheless, deciding which subgroup of patients actually require prompt empirical treatment still remains challenging. Indeed, there are no randomized controlled trials demonstrating the efficacy of empirical antifungal therapy on patient survival [36], thus limiting overall recommendations on timing. Moreover, empirical *Candida* treatment is frequently based on risk scores with very low positive predictive values that inevitably lead to unnecessary, expensive and sometimes toxic antifungal administration [37]. Despite such controversies, clinicians should be aware that empirical antifungals remain a common practice [38, 39]. Accordingly, when antifungals are prescribed empirically, it is critical to reassess the need for antifungal therapy 72–96 h after starting the treatment, especially when the initial diagnosis was uncertain. *Candida* biomarkers (CAGTA, T2Candida and 1,3-β-D-glucan assay) have emerged to assist clinicians in de-escalating unnecessary empirical therapy [38, 39]. A strategy using biomarkers among patients receiving empirical antifungals demonstrated a high negative predictive value (97% for the entire population and 100% among ICU patients) [38], thus permitting the safe early discontinuation of empirical therapy.

Regarding other IFIs (e.g. invasive aspergillosis, mucormycosis), no consensus exists about the exact timeframe for starting empirical therapy outside of neutropenic patients [40]. However, due to the high mortality associated with these infections, we suggest that patients with specific risks for developing IFI other than invasive candidiasis, should receive empirical treatment upon clinical suspicion occurs, even if definitive proof of infection has not yet been obtained. Fungal cultures, a combination of serological biomarkers (galactomannan, *Aspergillus* PCR and 1,3-β-D-glucan assay) along with computed tomography, should always be performed and treatment should be revised and eventually withheld if the diagnosis of fungal infection is not confirmed [40].

### Resistance avoidance with antimicrobial de-escalation

Antimicrobial de-escalation (ADE) refers to early modification of empiric antimicrobial therapy in order to prevent the emergence of antimicrobial resistance by decreasing overall exposure to broad-spectrum agents. It is known that the risk of new resistance emergence increases for each day of additional exposure to antipseudomonal β-lactam antibiotics ranging from 2% for meropenem to 8% for cefepime or piperacillin/tazobactam [41]. ADE is generally achieved by switching from combination antibiotics to monotherapy or by reducing the antimicrobial spectrum when broad-spectrum antibiotics are initially prescribed [42]. Additionally, reducing the number of administered antibiotics also offers the advantage of potentially reducing side effects and costs.

Many clinicians still are reluctant to modify initial broad-spectrum antibiotic regimens even when the practice is supported clinically and by microbiologic testing. To date, most studies have agreed on the fact that ADE is safe [42, 43]. One multicenter non-blinded trial of ADE compared to continued broad-spectrum therapy did find no difference in mortality but longer length of ICU stay in the ADE arm [44]. Among critically ill patients with proven candidemia, de-escalation from an echinocandin to fluconazole based on susceptibility testing was also found to be safe in terms of mortality and other outcomes [45]. Despite these data, the overall utilization of de-escalation is still low. In a recent multinational observational study (DIANA study), empirical therapy was de-escalated in only 16% of patients receiving initial broad-spectrum therapy [46]. Previous studies have reported ADE rates between 25 and 80% where the higher rates are generally reported from single centers focused on de-escalation for specific pathogens [43, 47–49].

The impact of ADE on resistance prevention has not been consistently demonstrated. In fact, few studies have specifically analyzed the effect of ADE on new antimicrobial resistance. One retrospective study of ADE did not find any prevention for the subsequent isolation of multi-drug resistant (MDR) pathogens in surveillance cultures or in ICU-acquired infections [50]. Montravers and colleagues also did not find a reduction of the emergence of MDR pathogens in a cohort of critically ill patients with intra-abdominal infections [51]. Similarly, the emergence of antibiotic-resistant bacteria was not reduced with de-escalation of empirical anti-pseudomonal beta-lactams in a retrospective study focused on the occurrence of new antibiotic resistance [52].

The DIANA study also did not demonstrate significant differences in the emergence of MDR pathogens following ADE [46]. However, emergence of MDR pathogens was numerically lower with ADE than in patients in whom empirical therapy was maintained (7.5% vs 11.9%; *p* = 0.052). Importantly, this study was not designed to draw definite conclusions about resistance emergence. In non-critically ill patients, a retrospective study that evaluated the safety of de-escalation of empiric carbapenems prescribed in an ESBL-endemic setting observed a significantly lower incidence of carbapenem-resistant *A. baumannii* acquisition in the group that underwent ADE [49]. The rate of adverse drug reactions was also significantly lower in the de-escalated group.

ADE is clearly feasible to carry out for both bacterial and fungal infections. ADE is safe and has been a recommended strategy in critically ill patients endorsed by an international position paper [42]. Clinicians should attempt to routinely carry out ADE focusing on the clinical response of the patient and the results of susceptibility testing. The use of appropriate antimicrobial doses and infusion durations will also help insure appropriate pharmacokinetic (PK) antibiotic exposure to optimize clinical outcomes.

### Antibiotic infusion duration to optimize drug pharmacokinetics

In addition to delivering timely appropriate antibiotic regimens, adequate drug concentrations at the infection site are needed to optimize clinical outcomes. The DALI study, a prospective, multicenter study, was primarily conducted to describe the frequency with which PK/pharmacodynamic (PK/PD) end points for β-lactam antibiotics were achieved in critically ill patients [53]. Achievement of PK/PD targets was highly variable among the different antibiotics studied, ranging from 35.0% for an aggressive target (100%* T*_FREE_ > 4 × MIC) to 78.9% for a traditionally acceptable target (50% *T*_FREE_ > MIC). These data suggest that many critically ill patients have inadequate antibiotic exposure as assessed by PK/PD endpoints.

Many factors influence the PK of antibiotics in critically ill patients and may contribute to subtherapeutic exposures. Hypoalbuminemia, large-volume crystalloid administration, large pleural effusions or abdominal ascites that increase the volume of distribution for hydrophilic drugs, catecholamines, and renal replacement therapies can all significantly alter infection site concentrations of administered antibiotics [54]. Another factor worth specific mention is augmented renal clearance (ARC). ARC is defined as a creatinine clearance (CrCl) greater than 130 mL/min/1.73 m^2^ in males and greater than 120 mL/min/1.73 m^2^ in females [55]. ARC has been linked with subtherapeutic β-lactam and glycopeptide concentrations [56, 57]. However, results have been conflicting in studies attempting to associate ARC with worse clinical outcomes [58–60]. ARC was implicated as a possible cause of treatment failure in a randomized controlled trial comparing 10 days of imipenem/cilastatin with 7 days of doripenem for ventilator-associated pneumonia caused by GNB [61]. Altogether, the study was terminated early because clinical cure rates were lower and mortality rates were higher in the doripenem group than in the imipenem group. Of interest, the largest difference in clinical cure rates was in the subgroup of patients with a CrCl greater than 150 mL/min/1.73 m^2^ [61].

The most common strategy studied to adjust for altered PK parameters in critically ill patients and achieve greater time above the MIC has been prolonged or continuous infusions of time-dependent antimicrobials, including β-lactams, carbapenems, and vancomycin. While numerous observational studies have shown better clinical cure rates with prolonged or continuous infusion of β-lactams, two meta-analyses have failed to confirm these findings [62, 63]. In contrast, a meta-analysis that included vancomycin and linezolid [64] and another that focused specifically on piperacillin/tazobactam or carbapenems [65] found improved clinical outcomes, including lower mortality, when antibiotics were administered by prolonged or continuous infusion compared with bolus injections.

The variability in outcomes between meta-analyses of prolonged or continuous antibiotic infusions is likely multifactorial but, in large part, a result of the lack of methodologic rigor and transparency as recommended by well-established standards for conducting such studies. Therefore the findings, both positive and negative, should be tempered by the presumed risk of bias [66]. It is also important to recognize that prolonged infusions of antibiotics will not compensate for poor initial drug selection, inferior drug characteristics, or underdosing of these agents in critically ill patients. The largest (*n* = 432) randomized, multicenter trial to date comparing continuous β-lactam infusions with intermittent infusions in critically ill patients with severe sepsis found no difference in alive ICU-free days, 90-day survival, or clinical cure 14 days after antibiotic cessation [67].

### Using AI/ML to improve sepsis outcomes

As the foundation of optimal sepsis care is fundamentally linked to the timing of key interventions, early recognition coupled with timely management strategies remain paramount to improving outcomes. Artificial Intelligence (AI) and Machine Learning (ML) are types of advanced mathematical models that combine computer science with statistical methods to yield highly accurate predictive models. These advanced computational tools can analyze enormous quantities of data to identify patterns from large, complex datasets. Sepsis, being a common entity with significant heterogeneity, combined with the large quantity of clinical data available, especially in the ICU, is a particularly attractive target for AI/ML-based analysis.

As a result, over the past 10 years, there has been a relative explosion in the use of AI/ML in sepsis, particularly around predicting onset time, which if done correctly, can help identify patients with impending sepsis and reduce time to appropriate antimicrobial therapy. One of the earliest approaches used a simple recursive partitioning and regression tree to identify ward patients who may become septic [68]. In this analysis, 70% of alerted patients had a sepsis-related intervention performed, suggesting the feasibility of early identification. This paved the way for additional analyses and in 2015, Henry and colleagues demonstrated that more advanced statistical tools could be combined with large, publicly available ICU databases, by creating a retrospective model that could predict septic shock (Sepsis-II criteria) 28.2 h (median) before onset with a sensitivity of 85% and specificity of 67% (area under the receiving operating characteristic curve [AUROC] 0.82) [69]. In 2016, the same data was used to train a different model which could predict sepsis (Sepsis-II criteria) 3-h ahead of clinical onset with a sensitivity of 0.90 at specificity of 0.81 (AUROC 0.83) [70]. Since then, yet further progress to operationalize advanced AI/ML techniques have spawned additional analyses using more robust AI/ML algorithms yielding similar results [71–73]. Furthermore, these advanced approaches have yielded incremental improvements in sepsis case recognition and prediction when compared to traditional early warning systems [72, 74]. Despite the promise of these retrospective models, only about 6% have been prospectively evaluated and when implemented have yielded mixed results on patient mortality and length of stay [68, 75, 76].

While rapid molecular diagnostic tests are increasingly being developed to identify pathogens and antibiotic resistance patterns, their cost and availability preclude widespread deployment. Similarly, even though these tests are considered “rapid”, they still require time for sample collection, lab delivery, and specimen analysis, during which time, antibiotic therapy is usually not withheld. AI/ML may be able to help bridge this time gap, by predicting antimicrobial resistance patterns, further facilitating antimicrobial stewardship. In a recent analyses, McGuire and colleagues demonstrated that longitudinal clinical data could be harmonized to predict the risk of carbapenem resistance [77]. In this investigation, new carbapenem resistant infections accounted for 1.6% of the population, yet the predictive model generated a sensitivity of 30%, a positive predictive value of 30% and a negative predictive value of 99% (AUROC 0.84). While AI/ML certainly cannot replace the role of rapid molecular testing, it may be able to facilitate upfront appropriate antimicrobial selection.

Beyond using AI/ML to predict onset time and antimicrobial resistance patterns, advancements in decision modeling are creating avenues for investigators to develop AI/ML algorithms to help determine optimal timing for fluid resuscitation and vasopressor initiation [78]. In this study, Komorowski and colleagues use a reinforcement model to learn optimal intravenous fluid resuscitation and vasopressor dosing strategies. Retrospective validation of this model revealed that mortality was the lowest when clinician actions matched the AI-based recommendations.

As we look towards the future of AI/ML in sepsis care, there are many necessary barriers that need to be overcome before wide scale deployment is achieved. These include the need for larger, more integrated datasets, a harmonized definition of sepsis suitable for automated extraction, more robust explainability, and prospective algorithm validation with emphasis on end-user needs, expectations and clinical workflows [79–81].

## Conclusions

**Fig. 3:**
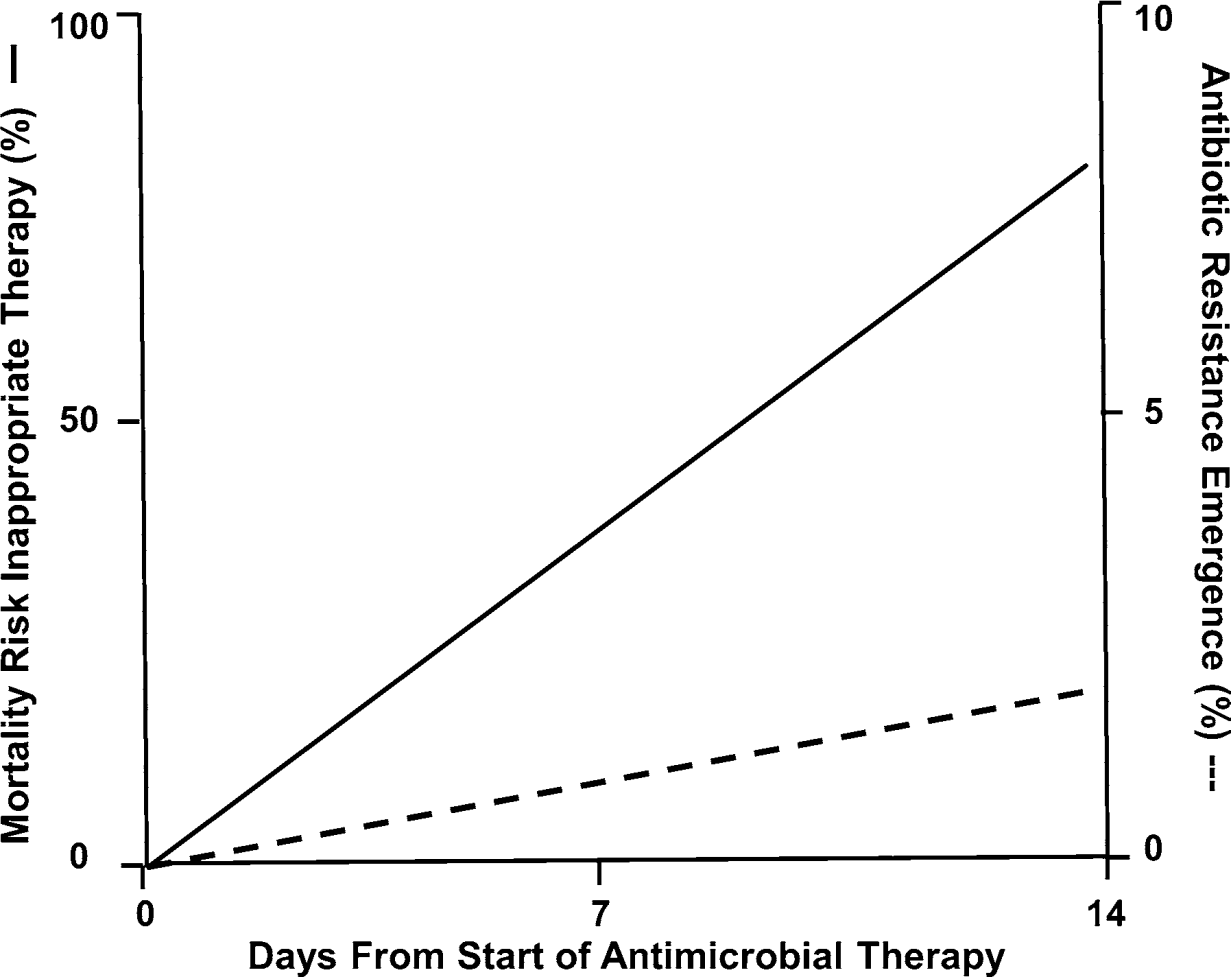
Solid line depicts increasing risk of mortality for each day that inappropriate antibiotic therapy is continued from the start of treatment. Dash line depicts increasing risk of new antibiotic resistance emergence for each day that antibiotic treatment is continued from the start of treatment

Time variables play an important role in the care of patients with life threatening infections. As Fig. 3 demonstrates, delaying appropriate antibiotic therapy increases the risk of death. At the same time, the risk of antibiotic resistance increases as the duration of antibiotic therapy is prolonged without a ceiling effect [41, 82]. Given these competing clinical outcomes, infection cure versus resistance emergence, clinicians must employ strategies that optimize their use of antimicrobials in the ICU. Table 1 provides some “common sense” recommendations that will assist clinicians in achieving a more harmonious balance in the ICU in regards to antibiotic utilization and timing. Future advances in non-antibiotic therapies for serious infections, rapid molecular diagnostics, and AI/ML should further enhance antibiotic timing practices in the ICU and improve patient outcomes while minimizing the use of unnecessary antimicrobial therapy.

**Table 1:** Summary and key recommendations

1.	Timing of antibiotic therapy is an important determinant of outcome especially in patients with septic shock and other life threatening infections. Numerous clinical studies have demonstrated that delaying the administration of antibiotic therapy, even by an hour or two in septic shock, can be associated with greater risk of death
2.	Ideally antimicrobial therapy should be initiated within three to five hours after infection onset in hospitalized patients, but immediately if possible when septic shock is present. All efforts should be taken to avoid administrative barriers to achieving this goal at the local hospital level
3.	The recommendations on timing of antimicrobial therapy appear similar regardless of the offending pathogen. However, the relationship between timing of antimicrobial administration and outcome has been best established in the medical literature for patients with bacterial and fungal infections, especially bloodstream infections and septic shock
4.	In addition to the timely administration of antibiotic therapy, careful consideration must be given to achieving timely source control of the infection including the removal of infected hardware (e.g., central venous catheters, intravascular ports) and drainage of infected fluid collections. Timely source control will allow for optimal antibiotic efficacy by lessening the influence of pathogen density in order to better achieve both clinical and microbiologic resolution of the infection
5.	Timely administration of antibiotics for life threatening infections must also include consideration for the selection of appropriate antimicrobial therapy (i.e., an antimicrobial regimen that is demonstrated to have in vitro activity against the offending pathogens causing the infection). Many observational clinical studies, both retrospective and prospective as well as one randomized trial in patients with Gram negative bacterial bloodstream infections, have demonstrated that the delayed administration of an appropriate antimicrobial regimen is associated with increased mortality. The association between delayed administration of an appropriate antimicrobial regimen and increased mortality has been shown for both community and hospital acquired infections, sepsis, septic shock, bloodstream infections, and nosocomial pneumonia
6.	Prolonged infusion times of β-lactam antibiotics over three to four hours, as opposed to infusion times of less than one hour, should be considered as an adjunctive approach towards improving antibiotic efficacy and reducing the propensity for the emergence of antibiotic resistance. Although, the available literature is mixed on the overall benefit of prolonged β-lactam infusions, there appears to be little likelihood for any increased adverse effects from prolonged infusion times
7.	Timely de-escalation of empiric broad-spectrum antimicrobial regimens given to achieve appropriate treatment of life threatening infections should occur based on available microbiology results and the clinical response of the patient. Avoidance of unnecessarily prolonged administration of broad-spectrum agents, based on available pathogen identification and susceptibility testing, should be routinely performed as the risk of resistance emergence increases incrementally with each day of antibiotic administration without a demonstrable ceiling effect
8.	With advances in molecular diagnostics for infectious diseases and machine learning/artificial intelligence algorithms for the prediction of infection occurrence, as well as the etiology of infection, achieving both more timely administration of appropriate antimicrobial therapy and lower propensity for resistance emergence should become more feasible. Future research should be directed at advancing these approaches for the care of infected critically ill patients

## Data Availability

Data sharing is not applicable to this article as no datasets were generated or analyzed during the current study.

## References

[1] Vincent JL, Sakr Y, Singer M, Martin-Loeches I, Machado FR, Marshall JC (2020). Prevalence and outcomes of infection among patients in intensive care units in 2017. JAMA.

[2] World Health Organization Report. https://www.who.int/antimicrobial-resistance/interagency-coordination-group/final-report/en/. Accessed 26 July 2021 2020.

[3] Wellcome Trust, United Kingdom Department of Health. 2014. Review on Antimicrobial Resistance. https://amr-review.org/. Accessed 26 Sep 2021.

[4] Centers for Disease Control and prevention. Antibiotic Resistance: A Global Threat. https://www.cdc.gov/drugresistance/solutions-initiative/stories/ar-global-threat.html Accessed 26 July 2021.

[5] Kollef MH, Sherman G, Ward S, Fraser VJ (1999). Inadequate antimicrobial treatment of infections: a risk factor for hospital mortality among critically ill patients. Chest.

[6] Lodise TP, Patel N, Kwa A, Graves J, Furuno JP, Graffunder E (2007). Predictors of 30-day mortality among patients with *Pseudomonas aeruginosa* bloodstream infections: impact of delayed appropriate antibiotic selection. Antimicrob Agents Chemother.

[7] Kollef M, Micek S, Hampton N, Doherty JA, Kumar A (2012). Septic shock attributed to Candida infection: importance of empiric therapy and source control. Clin Infect Dis.

[8] Zasowski EJ, Claeys KC, Lagnf AM, Davis SL, Rybak MJ (2016). Time is of the essence: the impact of delayed antibiotic therapy on patient outcomes in hospital-onset enterococcal bloodstream infections. Clin Infect Dis.

[9] Harris PNA, Tambyah PA, Lye DC, Mo Y, Lee TH, Yilmaz M (2018). Effect of piperacillin-tazobactam vs meropenem on 30-day mortality for patients with *E coli* or *Klebsiella pneumoniae* bloodstream infection and ceftriaxone resistance: a randomized clinical trial. JAMA.

[10] Kumar A, Roberts D, Wood KE, Light B, Parrillo JE, Sharma S (2006). Duration of hypotension before initiation of effective antimicrobial therapy is the critical determinant of survival in human septic shock. Crit Care Med.

[11] Seymour CW, Gesten F, Prescott HC, Friedrich ME, Iwashyna TJ, Phillips GS (2017). Time to treatment and mortality during mandated emergency care for sepsis. N Engl J Med.

[12] Zilberberg MD, Shorr AF, Micek ST, Vazquez-Guillamet MC, Kollef MH (2014). Multi- drug resistance, inappropriate initial antibiotic therapy and mortality in Gram- negative severe sepsis and septic shock: a retrospective cohort study. Crit Care.

[13] Vazquez-Guillamet MC, Scolari M, Zilberberg MD, Shorr AF, Micek ST, Kollef MH (2014). Using the number needed to treat to assess appropriate antimicrobial therapy as a determinant of outcome in severe sepsis and septic shock. Crit Care Med.

[14] Bassetti M, Rello J, Blasi F, Goossens H, Sotjiu G, Tavoschi L (2020). Systematic review of the impact of appropriate versus inappropriate initial antibiotic therapy on outcomes of patients with severe bacterial infections. Int J Antimicrob Agents.

[15] Klein Klouwenberg PM, Cremer OL, van Vught LA, Ong DS, Frencken JF, Schultz MJ (2015). Likelihood of infection in patients with presumed sepsis at the time of intensive care unit admission: a cohort study. Crit Care.

[16] Webb BJ, Sorensen J, Jephson A, Mecham I, Dean NC (2019). Broad-spectrum antibiotic use and poor outcomes in community-onset pneumonia: a cohort study. Eur Respir J.

[17] Rhee C, Kadri SS, Dekker JP, Danner RL, Chen HC, Fram D (2020). Prevalence of antibiotic-resistant pathogens in culture-proven sepsis and outcomes associated with inadequate and broad-spectrum empiric antibiotic use. JAMA Netw Open.

[18] Timsit JF, Bassetti M, Cremer O, Daikos G, de Waele J, Kallil A (2019). Rationalizing antimicrobial therapy in the ICU: a narrative review. Intensive Care Med.

[19] van Vught LA, Klein Klouwenberg PM, Spitoni C, Scicluna BP, Wiewel MA, Horn J (2016). Incidence, risk factors, and attributable mortality of secondary infections in the intensive care unit after admission for sepsis. JAMA.

[20] Barbier F, Pommier C, Essaied W, Garrouste-Orgeas M, Schwebel C, Ruckly S (2016). Colonization and infection with extended-spectrum beta-lactamase-producing Enterobacteriaceae in ICU patients: what impact on outcomes and carbapenem exposure?. J Antimicrob Chemother.

[21] Barbier F, Bailly S, Schwebel C, Papazian L, Azoulay E, Kallel H (2018). Infection-related ventilator-associated complications in ICU patients colonised with extended-spectrum beta-lactamase-producing Enterobacteriaceae. Intensive Care Med.

[22] Andremont O, Armand-Lefevre L, Dupuis C, de Montmollin E, Ruckly S, Lucet JC (2020). Semi-quantitative cultures of throat and rectal swabs are efficient tests to predict ESBL-Enterobacterales ventilator-associated pneumonia in mechanically ventilated ESBL carriers. Intensive Care Med.

[23] Garrouste-Orgeas M, Timsit JF, Kallel H, Ben Ali A, Dumay MF, Paoli B (2001). Colonization with methicillin-resistant Staphylococcus aureus in ICU patients: morbidity, mortality, and glycopeptide use. Infect Control Hosp Epidemiol.

[24] Kerneis S, Visseaux B, Armand-Lefevre L, Timsit JF (2021). Molecular diagnostic methods for pneumonia: how can they be applied in practice?. Curr Opin Infect Dis.

[25] Peri AM, Stewart A, Hume A, Irwin A, Harris PNA (2021). New microbiological techniques for the diagnosis of bacterial infections and sepsis in ICU including point of care. Curr Infect Dis Rep.

[26] Ripa M, Rodriguez-Nunez O, Cardozo C, Naharro-Abellan A, Almela M, Marco F (2017). Influence of empirical double-active combination antimicrobial therapy compared with active monotherapy on mortality in patients with septic shock: a propensity score-adjusted and matched analysis. J Antimicrob Chemother.

[27] Gutierrez-Gutierrez B, Salamanca E, de Cueto M, Hsueh PR, Viale P, Pano-Pardo JR (2017). Effect of appropriate combination therapy on mortality of patients with bloodstream infections due to carbapenemase-producing Enterobacteriaceae (INCREMENT): a retrospective cohort study. Lancet Infect Dis.

[28] Andes DR, Safdar N, Baddley JW, Playford G, Reboli AC, Rex JH (2012). Impact of treatment strategy on outcomes in patients with candidemia and other forms of invasive candidiasis: a patient-level quantitative review of randomized trials. Clin Infect Dis.

[29] Morrell M, Fraser VJ, Kollef MH (2005). Delaying the empiric treatment of candida bloodstream infection until positive blood culture results are obtained: a potential risk factor for hospital mortality. Antimicrob Agents Chemother.

[30] Garey KW, Rege M, Pai MP, Mingo DE, Suda KJ, Turpin RS (2006). Time to initiation of fluconazole therapy impacts mortality in patients with candidemia: a multi-institutional study. Clin Infect Dis.

[31] Bassetti M, Vena A, Russo A (2018). Management of patients with septic shock due to Candida infection. Hosp Pract.

[32] Bassetti M, Righi E, Ansaldi F, Merelli M, Scarparo C, Antonelli M (2015). A multicenter multinational study of abdominal candidiasis: epidemiology, outcomes and predictors of mortality. Intensive Care Med.

[33] Bassetti M, Righi E, Ansaldi F, Merelli M, Trucchi C, De Pascale G (2014). A multicenter study of septic shock due to candidemia: outcomes and predictors of mortality. Intensive Care Med.

[34] Grim SA, Berger K, Teng C, Gupta S, Layden JE, Janda WM (2012). Timing of susceptibility-based antifungal drug administration in patients with Candida bloodstream infection: correlation with outcomes. J Antimicrob Chemother.

[35] Pappas PG, Kauffman CA, Andes DR, Clancy CJ, Marr KA, Ostrosky-Zeichner L (2016). Executive summary: clinical practice guideline for the management of Candidiasis: 2016 update by the infectious diseases Society of America. Clin Infect Dis.

[36] Timsit JF, Azoulay E, Schwebel C, Charles PE, Cornet M, Souweine B (2016). Empirical micafungin treatment and survival without invasive fungal infection in adults with ICU-acquired sepsis, candida colonization, and multiple organ failure: The EMPIRICUS randomized clinical trial. JAMA.

[37] Martin-Loeches I, Antonelli M, Cuenca-Estrella M, Dimopoulos G, Einav S, De Waele J (2019). ESICM/ESCMID task force on practical management of invasive candidiasis in critically ill patients. Intensive Care Med.

[38] Martinez-Jimenez MC, Munoz P, Valerio M, Vena A, Guinea J, Bouza E (2015). Combination of Candida biomarkers in patients receiving empirical antifungal therapy in a Spanish tertiary hospital: a potential role in reducing the duration of treatment. J Antimicrob Chemother.

[39] Munoz P, Vena A, Machado M, Gioia F, Martinez-Jimenez MC, Gomez E (2018). T2Candida MR as a predictor of outcome in patients with suspected invasive candidiasis starting empirical antifungal treatment: a prospective pilot study. J Antimicrob Chemother.

[40] Bassetti M, Peghin M, Vena A (2018). Challenges and solution of invasive aspergillosis in non-neutropenic patients: a review. Infect Dis Ther.

[41] Teshome BF, Vouri SM, Hampton N, Kollef MH, Micek ST (2019). Duration of exposure to antipseudomonal β-lactam antibiotics in the critically ill and development of new resistance. Pharmacotherapy.

[42] Tabah A, Bassetti M, Kollef MH, Zahar J-R, Paiva J-A, Timsit J-F (2020). Antimicrobial de-escalation in critically ill patients: a position statement from a task force of the European Society of Intensive Care Medicine (ESICM) and European Society of Clinical Microbiology and Infectious Diseases (ESCMID) Critically Ill Patients Study Group (ESGCIP). Intensive Care Med.

[43] Tabah A, Cotta MO, Garnacho-Montero J, Schouten J, Roberts JA, Lipman J (2016). A systematic review of the definitions, determinants, and clinical outcomes of antimicrobial de-escalation in the intensive care unit. Clin Infect Dis.

[44] Leone M, Bechis C, Baumstarck K, Lefrant J-Y, Albanèse J, Jaber S (2014). De-escalation versus continuation of empirical antimicrobial treatment in severe sepsis: a multicenter non-blinded randomized noninferiority trial. Intensive Care Med.

[45] Garnacho-Montero J, Díaz-Martín A, Cantón-Bulnes L, Ramírez P, Sierra R, Arias-Verdú D (2018). Initial antifungal strategy reduces mortality in critically ill patients with candidemia: a propensity score-adjusted analysis of a multicenter study. Crit Care Med.

[46] De Bus L, Depuydt P, Steen J, Dhaese S, De Smet K, Tabah A (2020). Antimicrobial de-escalation in the critically ill patient and assessment of clinical cure: the DIANA study. Intensive Care Med.

[47] Heenen S, Jacobs F, Vincent J-L (2012). Antibiotic strategies in severe nosocomial sepsis: why do we not de-escalate more often?. Crit Care Med.

[48] Garnacho-Montero J, Gutiérrez-Pizarraya A, Escoresca-Ortega A, Corcia-Palomo Y, Fernández-Delgado E, Herrera-Melero I (2014). De-escalation of empirical therapy is associated with lower mortality in patients with severe sepsis and septic shock. Intensive Care Med.

[49] Lew KY, Ng TM, Tan M, Tan SH, Lew EL, Ling LM (2015). Safety and clinical outcomes of carbapenem de-escalation as part of an antimicrobial stewardship programme in an ESBL-endemic setting. J Antimicrob Chemother.

[50] Gonzalez L, Cravoisy A, Barraud D, Conrad M, Nace L, Lemarié J (2013). Factors influencing the implementation of antibiotic de-escalation and impact of this strategy in critically ill patients. Crit Care.

[51] Montravers P, Augustin P, Grall N, Desmard M, Allou N, Marmuse J-P (2016). Characteristics and outcomes of anti-infective de-escalation during health care-associated intra-abdominal infections. Crit Care.

[52] De Bus L, Denys W, Catteeuw J, Gadeyne B, Vermeulen K, Boelens J (2016). Impact of de-escalation of beta-lactam antibiotics on the emergence of antibiotic resistance in ICU patients: a retrospective observational study. Intensive Care Med.

[53] Roberts JA, Paul SK, Akova M, Bassetti M, De Waele JJ, Dimopoulos G (2014). DALI: defining antibiotic levels in intensive care unit patients: are current β-lactam antibiotic doses sufficient for critically ill patients?. Clin Infect Dis.

[54] Veiga RP, Paiva JA (2018). Pharmacokinetics-pharmacodynamics issues relevant for the clinical use of beta-lactam antibiotics in critically ill patients. Crit Care.

[55] Cook AM, Hatton-Kolpek J (2019). Augmented renal clearance. Pharmacotherapy.

[56] Carlier M, Carrette S, Roberts JA, Stove V, Verstraete A, Hoste E (2013). Meropenem and piperacillin/tazobactam prescribing in critically ill patients: does augmented renal clearance affect pharmacokinetic/pharmacodynamic target attainment when extended infusions are used?. Crit Care.

[57] Baptista JP, Sousa E, Martins PJ (2012). Augmented renal clearance in septic patients and implications for vancomycin optimisation. Int J Antimicrob Agents.

[58] Claus BO, Hoste EA, Colpaert K, Robays H, Decruyenaere J, De Waele JJ (2013). Augmented renal clearance is a common finding with worse clinical outcome in critically ill patients receiving antimicrobial therapy. J Crit Care.

[59] Udy AA, Dulhunty JM, Roberts JA, Davis JS, Webb SAR, Bellomo R (2017). Association between augmented renal clearance and clinical outcomes in patients receiving beta-lactam antibiotic therapy by continuous or intermittent infusion: a nested cohort study of the BLING-II randomised, placebo-controlled trial. Int J Antimicrob Agents.

[60] Burnham JP, Micek ST, Kollef MH (2017). Augmented renal clearance is not a risk factor for mortality in Enterobacteriaceae bloodstream infection treated with appropriate empiric antimicrobials. PLoS ONE.

[61] Kollef MH, Chastre J, Clavel M, Restrepo MI, Michiels B, Kaniga K (2012). A randomized trial of 7-day doripenem versus 10-day imipenem-cilastatin for ventilator-associated pneumonia. Crit Care.

[62] Roberts JA, Webb S, Paterson D, Ho KM, Lipman J (2009). A systematic review on clinical benefits of continuous administration of beta-lactam antibiotics. Crit Care Med.

[63] Kasiakou SK, Sermaides GJ, Michalopoulos A, Soteriades ES, Falagas ME (2005). Continuous versus intermittent intravenous administration of antibiotics: a meta-analysis of randomised controlled trials. Lancet Infect Dis.

[64] Chant C, Leung A, Friedrich JO (2013). Optimal dosing of antibiotics in critically ill patients by using continuous/extended infusions: a systematic review and meta-analysis. Crit Care.

[65] Falagas ME, Tansarli GS, Ikawa K, Vardakas KZ (2013). Clinical outcomes with extended or continuous versus short-term intravenous infusion of carbapenems and piperacillin/tazobactam: a systematic review and meta-analysis. Clin Infect Dis.

[66] Thabet P, Joshi A, MacDonald E, Hutton B, Cheng W, Stevens A (2021). Clinical and pharmacokinetic/dynamic outcomes of prolonged infusions of beta-lactam antimicrobials: an overview of systematic reviews. PLoS ONE.

[67] Dulhunty JM, Roberts JA, Davis JS (2015). A multicenter randomized trial of continuous versus intermittent β-lactam infusion in severe sepsis. Am J Respir Crit Care Med.

[68] Sawyer AM, Deal EN, Labelle AJ, Witt C, Thiel SW, Heard H (2011). Implementation of a real-time computerized sepsis alert in nonintensive care unit patients. Crit Care Med.

[69] Henry KE, Hager DN, Pronovost PJ, Saria S (2015). A targeted real-time early warning score (TREWScore) for septic shock [Internet]. Sci Transl Med..

[70] Calvert JS, Price DA, Chettipally UK, Barton CW, Feldman MD, Hoffman JL (2016). A computational approach to early sepsis detection. Comput Biol Med.

[71] Reyna MA, Josef CS, Jeter R, Shashikumar SP, Westover MB, Nemati S (2020). Early prediction of sepsis from clinical data: the PhysioNet/computing in cardiology challenge 2019. Crit Care Med.

[72] Fleuren LM, Klausch TLT, Zwager CL, Schoonmade LJ, Guo T, Roggeveen LF (2020). Machine learning for the prediction of sepsis: a systematic review and meta-analysis of diagnostic test accuracy. Intensive Care Med.

[73] Mao Q, Jay M, Hoffman JL, Calvert J, Barton C, Simabukuro D (2018). Multicentre validation of a sepsis prediction algorithm using only vital sign data in the emergency department, general ward and ICU. BMJ Open.

[74] Shivakumar N, Betthauser K, Gupta A, Lai A, Kollef MH, Payne P, et al. Comparison of Early warning scores for sepsis early identification and prediction in the general ward setting. JAMIA. 2021 (Epub ahead of print). 10.1093/jamiaopen/ooab062 PMC8607822 34820600

[75] Shimabukuro DW, Barton CW, Feldman MD, Mataraso SJ, Das R (2017). Effect of a machine learning-based severe sepsis prediction algorithm on patient survival and hospital length of stay: a randomised clinical trial. BMJ Open Respir Res.

[76] Giannini HM, Ginestra JC, Chivers C, Draugelis M, Hanish A, Schweickert WD (2019). A machine learning algorithm to predict severe sepsis and septic shock: development, implementation, and impact on clinical practice. Crit Care Med.

[77] McGuire RJ, Yu SC, Payne PRO, Lai AM, Vazquez-Guillamet MC (2021). A pragmatic machine learning model to predict carbapenem resistance. Antimicrob Agents Ch.

[78] Komorowski M, Celi LA, Badawi O, Gordon AC, Faisal AA (2018). The artificial intelligence clinician learns optimal treatment strategies for sepsis in intensive care. Nat Med.

[79] Yu SC, Betthauser KD, Gupta A, Lyons PG, Lai AM, Kollef MH (2021). Comparison of sepsis definitions as automated criteria. Crit Care Med.

[80] Petersen C, Smith J, Freimuth RR, Goodman KW, Jackson GP, Kannry J (2021). Recommendations for the safe, effective use of adaptive CDS in the US healthcare system: an AMIA position paper. J Am Med Inform Assn.

[81] Saria S, Henry KE (2020). Too many definitions of sepsis. Crit Care Med.

[82] Teshome BF, Vouri SM, Hampton NB, Kollef MH, Micek ST (2020). Evaluation of a ceiling effect on the association of new resistance development to antipseudomonal beta-lactam exposure in the critically ill. Infect Control Hosp Epidemiol.

